# Obstacles of Online Learning Facing Nursing Students after the COVID-19 Pandemic

**DOI:** 10.1155/2024/5387908

**Published:** 2024-04-22

**Authors:** Haitham Khatatbeh, Murad Alkhalaileh, Ibrahim Ayasreh, Atallah Habahbeh, Laith Alosoufe, Nisser Alhroub, Mohammed Albashtawy, Manar Abu-Abbas, Tariq Al-Dwaikat, Amira Mohammed Ali

**Affiliations:** ^1^Faculty of Nursing, Jerash University, Jerash, Jordan; ^2^Princess Salma Faculty of Nursing, Al Al-Bayt University, Al-Mafraq, Jordan; ^3^Department of Community and Mental Health, Faculty of Nursing, Jordan University of Science and Technology, Irbid, Jordan; ^4^Department of Psychiatric Nursing and Mental Health, Faculty of Nursing, Alexandria University, Smouha, Alexandria, Egypt

## Abstract

**Background:**

After the COVID-19 pandemic, the online style of instruction started to replace the traditional style in Jordan.

**Aims:**

This study aims to (1) assess the nursing students' perceived obstacles to online learning in Jordan; (2) explore significant relationships between participants' characteristics and their perceived obstacles; and (3) assess for significant differences in the perceived obstacles based on participants' characteristics.

**Methods:**

A cross-sectional, descriptive design was utilized in this study. A convenient sample of 325 nursing students responded to a self-reported questionnaire utilizing Google Forms. Both descriptive and inferential statistics were used to analyze the dataset using the SPSS software.

**Results:**

The mean scores of the obstacles to online learning were 2.94 (SD = 0.95) for the academic obstacles subscale, 2.90 (SD = 0.83) for the technological obstacles subscale, and 3.25 (SD = 1.00) for the administrative obstacles subscale. Significant associations were found between participants' characteristics and perceived obstacles to online learning. For instance, the type of university was significantly associated with academic (*r* = −0.32, *p* < 0.01), technological (*r* = −0.21, *p* < 0.01), and administrative obstacles (*r* = −0.32, *p* < 0.01). Furthermore, significant differences were found in the perceived obstacles based on the participants' demographic and studentship-related characteristics.

**Conclusions:**

According to their perceptions of online learning, nursing students in Jordan face three types of obstacles: academic, technical, and administrative. Decision-makers should intervene to enhance the online learning experience by overcoming the reported obstacles.

## 1. Introduction

Over the last two decades, online teaching has begun to spread in higher education, especially in the United States [1]. Despite the immense advancements in teaching technologies and Internet access, traditional teaching remains the dominant style of instruction in universities worldwide [2–4]. The reason behind this domination could be that face-to-face teaching has several advantages such as the high and immediate interaction between students and teachers, the less distractive environment, and socialization between students and teachers and between students themselves, especially in the small lecture halls. However, face-to-face teaching has several disadvantages including traveling costs and time rigidity [5].

In 2019, the coronavirus disease (COVID-19) started to spread around the world causing millions of deaths [6]. Therefore, the World Health Organization (WHO) recommended governments around the world to implement rapid responses to the COVID-19 pandemic [7]. In response to WHO recommendations, governments restricted traveling, closed airports, applied social distancing measures [8], and used online teaching as an alternative to face-to-face teaching [3].

Indeed, the recent pandemic has tipped the scales and driven higher education institutions to adopt the online style of instruction [2, 4, 5, 9–12]. Despite its advantages, such as time flexibility [4, 5] and lower costs [4, 11], the online style of instruction has faced many hurdles [2, 4]. These hurdles include poor Internet access [4, 9], lack of student and teacher coaching [13], a disruptive household setting [2, 14], and scarce digital backing [11, 15], which are the most pressing issues confronted by teachers and students. The root cause for these barriers could be the unexpected and unplanned use as a result of the COVID-19 pandemic [11].

Following the epidemic, a lot of colleges around the world embraced the blended/online style of instruction [10]. The blended style of instruction is described as a mixture of conventional and electronic teaching [16, 17]. This kind of teaching gives students the freedom of electronic learning while still allowing them to connect with their peers and teachers in a typical classroom setting [16, 18]. Another rationale for using this approach is to stay up to date with the emerging trend of combining electronic and traditional instruction [18, 19]. However, there are some issues that institutions should keep in mind when introducing the hybrid approach. First is ensuring that the quality of blended courses is comparable to that of traditional courses. Second, universities must ensure that students and instructors have access to the necessary resources, training, and support to succeed in integrating the blended style [3, 20, 21].

The negative attitude towards online teaching and lack of previous experience in online teaching should not be overlooked [11]. Some of the reported obstacles in online teaching are related to session control such as controlling student participation [11]. Another study found that students feel stressed, complain of poor sound clarity, and fail to learn in the home atmosphere [15].

According to a Saudi Arabian study, obstacles facing both students and teachers in online teaching can be academic, technical, or managerial. Academic obstacles for teachers include the time needed to prepare online teaching materials, the absence of communication with students, and the time needed to prepare online exams. Academic obstacles for students include the absence of communication with teachers, unavailability of time needed to complete the online course requirements, and unapproachable online course materials. Technical obstacles for teachers include a lack of technical backing, unavailable technological requirements and training, and the difficult use of online teaching software. For students, technical obstacles include the difficult use of online teaching software, and the unavailable technological requirements, training, and technical backing. Managerial obstacles for teachers include poor managerial reassurance, weak Internet access, negative criticism, and poor online teaching infrastructure. For students, managerial obstacles include weak Internet access, negative criticism, and poor online teaching infrastructure [9].

To date, the immature technical infrastructure of online teaching is a key obstacle, especially in third-world countries such as Jordan. For example, statistical reports indicate that the percentage of Internet users in Jordan is only 66% compared with 91% of United States inhabitants and 98% of Saudi Arabia inhabitants [22, 23]. Due to such circumstances, online teaching is still at its beginning at Jordanian universities. Taking into consideration these inputs, obstacles that hinder online teaching in Jordan are expected to be more impactful. A Jordanian study assessed students' perceived barriers to online teaching before the COVID-19 pandemic [24] and found that students were concerned about online teaching infrastructure and effectiveness, and whether they were delighted to use it. Furthermore, the case is even more complicated with health sciences teaching such as nursing teaching. The reason behind this particularity is that nursing students need to demonstrate practical skills and undergo clinical training [13, 25–27].

Jordan is one of the developing Middle Eastern countries that incorporated the online style of instruction in higher education institutions after the COVID-19 pandemic [28]. Despite the immense advancements in teaching technologies and Internet access around the world, students started to face several obstacles in using online learning during and after the COVID-19 pandemic [9, 29]. It is essential for universities to carefully consider these obstacles and develop strategies to overcome them.

In Jordan, obstacles to online learning facing nursing students are not yet well understood whether they are academic, administrative, or technical. To the best of our knowledge, no study yet has addressed the obstacles of online learning among nursing students, particularly in Jordan. This means that the current study is the first Jordanian study to evaluate difficulties facing online learning among nursing students. Knowing these obstacles might help decision-makers in higher education to ease the way of online learning by making appropriate laws. Thus, the main aim of this study was to identify obstacles to online learning facing nursing students after the COVID-19 pandemic in Jordan. The second aim was to search for significant relationships between participants' characteristics and perceived obstacles to online learning. The third aim of this study was to look for significant variances in the perceived obstacles to online learning based on participants' characteristics.

## 2. Methods

### 2.1. Design

This study utilized a cross-sectional descriptive design.

### 2.2. Sample

The sample was selected conveniently by using the snowball technique in which researchers invited nursing students at Jerash University to participate in this study. Then, the invited participants were asked to invite nursing students from other universities in Jordan. To reduce the potential bias with our convenient sample, the researchers targeted key traits for our target population (nursing students in Jordan). These traits included gender, year of study, type of university (public/private), and type of study (regular undergraduate students/diploma-to-bachelor bridging students). To avoid these traits being underrepresented, the researchers were keen to invite students from different academic levels. For example, the researchers selected students from basic and advanced courses to represent new and old students, respectively. In addition, two of the research teams who work at the governmental universities did the same at their institutions to represent the targeted traits.

To guarantee a satisfactory statistical power, a priori sample size calculation was performed using G*∗*Power software 3.1 [30]. Using the *t*-test approach/Wilcoxon–Mann–Whitney test with a medium effect size, significance set at 0.05, and power at 0.95, a sample size of 184 was needed. However, the post hoc analysis showed that our final sample with 325 participants provided a power of 0.99.

### 2.3. Data Collection

In this study, data were collected online using Google Forms® in the period between 17 January and 31 March 2023. A link to the questionnaire was created and shared with the potential participants at Jordanian universities. Those students who agreed to participate in this study were voluntarily asked to fill out a validated questionnaire. To reduce the potential selection bias by using Google Forms®, the researchers distributed the link through multiple channels to reach different groups and represent different participants' traits. The channels included WhatsApp®, Facebook®, and e-mail addresses.

### 2.4. Instrument

Participants responded to a group of demographics and studentship-related characteristics, and a validated questionnaire on obstacles to online learning. This instrument is a validated questionnaire composed of 11 Likert items (5 strongly agree and 1 strongly disagree) measuring three dimensions namely: academic (three items), technological (five items), and administrative obstacles (three items) of online learning [9]. According to Ja'ashan [9], Cronbach's alpha for the instrument was 0.8.

### 2.5. Ethical Consideration

As this study involved human subjects, ethical approval was obtained from the Institutional Review Board (IRB) at Jerash University according to the decision no. 3/4/2022/2023. Regarding the instrument use, permission was obtained from the instrument creator. Voluntary participation and free withdrawal from the study were ensured on the study's coversheet.

### 2.6. Data Analysis

The Statistical Package for the Social Sciences (SPSS) software was used in the data analysis. After downloading our data from Google Forms as an Excel sheet, the data were copied to the SPSS, cleaned, and coded. First, Kolmogorov–Smirnov and Shapiro–Wilk tests were used to check whether the data were normally distributed and to select statistical tests accordingly.

Kolmogorov–Smirnov and Shapiro–Wilk tests showed that the three subscales' data were nonnormally distributed and that the overall score's data were normally distributed. To normalize our data by removing the outliers, the square root transformation technique was used. As it did not work, we used nonparametric statistics.

Statistical tests included frequencies and percentages for categorical variables such as gender and marital status. In addition, mean and standard deviation were used to describe participants' scores on the outcome variables (academic, technological, and administrative obstacles and overall score). Spearman's correlation was used to find the significant relationships among variables.

To look for significant differences in the outcome variables based on participants' demographic and studentship-related characteristics, the Mann–Whitney test (for variables with two groups) and the Kruskal–Wallis test (for variables with three or more groups) were used. A *p* value of less than 0.05 was considered statistically significant.

## 3. Results

### 3.1. Scale Reliability

In this study, the internal consistency of Cronbach's alpha coefficient was used to assess the reliability of the questionnaire. According to the results of the scales, Cronbach's alpha coefficient for the scale used was 0.872. However, Cronbach's alpha coefficient for the three subscales was 0.653 for the academic subscale, 0.776 for the technological subscale, and 0.777 for the administrative subscale.

### 3.2. Participants' Characteristics and Scores on Outcome Variables

This study collected data from 325 participants in Jordan, of which 226 (69.5%) were female, 245 (75.4%) were single, and 220 (67.7%) were from private universities. Regarding their geographical regions, 203 (62.5%) were from the northern region, 112 (34.5%) were from the middle region, and 10 (3.1%) were from the southern region.  Table 1 shows more details on participants' characteristics such as the study program, number of family members, family income, living place, study year, available Internet, and available electronic devices.

Regarding the participants' scores on the outcome variables, the mean overall score was 3.0 (±0.78). On the subscales, the lowest mean score was on the technological subscale (2.90 ± 0.83) and the highest mean score was on the administrative subscale (3.25 ± 1.0).

### 3.3. Correlations among Study Variables

As shown in Table 2, Spearman's correlation coefficients showed significant associations among the participants' demographic and studentship characteristics and their scores on the outcome variables. For instance, significant associations were found among available Internet options and available electronic devices with participants' scores on the academic and administrative subscales, as well as with the overall score. Also, participants' living style was associated with their score on the technological subscale and the overall score. In addition, significant relationships were found among the study program (bachelor or bridging) and type of university (private or governmental) with participants' scores on the three subscales and overall scores.

### 3.4. Differences in the Mean Overall Score Based on the Participants' Characteristics

*T*-test and ANOVA were appropriately used to find any differences in the overall score based on the participants' characteristics. As shown in Table 3, participants in the regular study program showed higher overall scores (3.23 ± 0.72) than those in the bridging program (2.79 ± 0.78). Interestingly, participants from governmental universities demonstrated higher overall scores (3.36 ± 0.65) than those from private universities (2.84 ± 0.79). Furthermore, married participants showed the lowest overall scores (2.66 ± 0.80) than single (3.12 ± 0.74) and divorced/married participants (32.72 ± 1.03). Likewise, participants living with their families scored a lower mean overall score (2.98 ± 0.78) than those living with colleagues (3.62 ± 0.8028) or at university dormitory (3.95 ± 0.61).

### 3.5. Differences in the Mean Subscales' Scores Based on the Participants' Characteristics

The Mann–Whitney test was used to find variations in the subscales' mean rank scores based on participants' gender, study program, and type of university (binary variables). As shown in Table 4, the results revealed no significant differences in the subscales' mean rank scores based on participants' gender. However, significant differences were found based on the study program and type of university. Participants in the bridging program showed lower mean rank scores on the three subscales than those in the regular program. In addition, participants from private universities demonstrated lower mean rank scores on the three subscales than those from governmental universities.

The Kruskal–Wallis test was used to find variations in the subscales' mean rank scores based on participants' characteristics in three or more groups. Results showed no significant differences in the subscales' mean rank scores based on total family income, geographical region, or study year. Nevertheless, results showed significant differences in the subscales' mean rank scores based on marital status, number of family members, living place, available Internet, and available electronic devices. For instance, married participants demonstrated the lowest mean rank scores on the two subscales (technological and administrative subscales) than their counterparts. On the academic subscale, they scored lower mean rank than single participants and higher mean rank than divorced/married participants.

Based on the number of family members, the only subscale that showed significant differences in the mean rank scores was the academic subscale. Those participants with less than five family members showed lower mean rank scores than those with five to nine family members. Interestingly, participants with 10 or more family members showed the lowest mean rank scores.

Participants living alone or with their families showed lower mean rank scores than those living in university dormitories or with their colleagues/friends on the three subscales (academic, technological, and administrative subscales).

Participants with no Internet access at their homes showed the highest mean rank scores than their counterparts, on the three subscales. Interestingly, participants with more than two sources on the Internet showed higher mean rank scores on the three subscales than those with one source.

Participants who have laptops showed the lowest mean rank scores on the three subscales than participants with mobile phones, tablets, or even desktop computers, as shown in Table 5.

## 4. Discussion

This study aimed to assess the students' perceived levels of academic, technical, and administrative obstacles to online learning facing nursing students in Jordan. Our results showed that the lowest mean score was on the technological subscale. This means that participants face low levels of technological obstacles. This finding can be explained by technological literacy, especially among educated people such as university students [31]. However, two items (fifth and seventh items) within the technological subscale had the highest mean score than other items within the same subscale. These items are related to a lack of technological support and a lack of training courses provided by the institution, and this matches an Indian study about the obstacles of online teaching during the last pandemic [32].

On the other hand, the highest mean score was on the administrative subscale. This means that the administrative obstacles are the biggest obstacles hindering online learning among the participants. The three items of the administrative subscale (ninth to eleventh items) showed relatively high mean scores. These items are related to problems with Internet access, negative comments on online learning, and inadequate infrastructure. Our explanation for this result might be the high bureaucracy in the third-world countries such as Jordan [33]. Our findings were different from those found in a Saudi study [9]; in their study, the highest mean score was for technological obstacles and the lowest mean score was for academic obstacles. These differences can be secondary to the different samples as they studied university students in the English department or to the different technological and academic abilities between Saudi and Jordanian universities.

Regarding the academic obstacles, the mean score of this subscale occupied the middle rank between the technological and administrative obstacle subscales. Within the academic subscale, two items (the first and second items) had relatively high mean scores. These items are related to the lack of interaction between students and teaching staff and the lack of time required to have exams/assignments. This finding can be explained by the nature of online learning which usually limits the teacher-student interaction [5].

The second aim of this study was to explore significant relationships among participants' demographic and studentship-related characteristics with perceived academic, technical, and administrative obstacles. This study showed significant associations between available Internet options and available electronic devices with participants' scores on the academic and administrative subscales, as well as with the overall score. This result reflects the well-known vital role of technology availability in facilitating online learning [9, 10].

In addition, our results showed significant relationships among the type of university (private or governmental) and the three subscales as well as the overall score. These significant relationships can be explained by several explanations. For instance, the different learning management systems used in each university might be different from each other in terms of simplicity. Another explanation is the higher academic level of students at governmental universities than at private universities. Furthermore, training on how to use these systems is also different between private and governmental universities [34].

This study aimed to examine significant differences in the perceived academic, technical, and administrative obstacles based on participants' demographic and studentship-related characteristics. Our results showed higher mean ranks for the three subscales and a higher mean for the overall score among students in bachelor programs than those in bridging programs. This finding looks logical as students in bridging programs are more experienced in online learning than their counterparts. In Jordan, students with a 2-year diploma can obtain a bachelor's degree through 2-year bridging programs. Thus, students in bridging programs were enrolled in a college, experienced in online learning, and are more intellectually mature than students in bachelor programs. This can make online learning among bridging students easier than their counterparts. This finding is supported by an Egyptian study which found that fourth-year students face the least difficulty in online learning [35].

Regarding the type of university, governmental university students showed higher mean ranks for the three subscales and a higher mean for the overall score among students in bachelor programs than those in private universities. This finding means that students at governmental universities in Jordan face more obstacles than those at private universities. This result can be explained by the higher quality of support provided by private universities than by government universities, as found by another study about factors that influence online learning in private universities [34]. In addition, our findings can be explained by the different economic classes of the students' families. In other words, relatively rich families send their children to private universities and provide them with technological means and Internet access. On the other hand, poor families might not be able to offer technological aid and send their children to government universities. Furthermore, it is believed that studying at private universities is much easier than at governmental universities and fewer requirements reduce the challenges. This finding is different from a Pakistani study which found no significant differences between public and private universities [36]. This difference might be related to the different economic strengths between Jordan, which is a middle-income country, and Pakistan, which is a low-income country [37].

In terms of marital status and living place, married students and those living with their families showed the lowest mean ranks for the three subscales and the lowest mean for the overall score than single students and those living far from their families. This result can be explained by the care and support the students receive from their spouses or families, compared with their single or living-alone counterparts. This explanation is supported by a systematic review which found that peer and family support is linked with student doggedness in distance education programs [38]. This finding might stress the importance of student family support during their studies.

Although this study has a good sample size, the results of this study are limited by several factors. For instance, the cross-sectional design and relatively homogenous sample can limit the generalization of results. Furthermore, the obstacles of online learning were studied only from the student's perspective without assessing teachers' perspectives. Future studies may study the obstacles of online learning internationally and assess teachers' perspectives.

## 5. Conclusion

Online learning has become an integral part of nursing learning around the world. However, there are still many obstacles that stand in the way of online learning in the nursing field. Participants of the current study faced several obstacles hindering their online learning. These obstacles could either be administrative, technological, or academic. Universities' academic decision-makers and stakeholders are advised to intervene to turn these obstacles into enablers. Interventions can primarily include orientation programs upon entrance and continuous training programs to guide nursing students on how to use online learning software. In addition, organizing social activities and encouraging the exchange of experiences could be beneficial, especially for students living far from their families. Furthermore, financial support could help students overcome technological obstacles if they are able to obtain the needed devices and Internet access. Finally, universities might make a borrowing system to provide electronic devices for needy students during their study period.

## Figures and Tables

**Table 1:** Participants' characteristics and scores on outcome variables (*N* = 325).

Characteristic	Frequency	Percentage

*Gender*		
Male	99	30.5
Female	226	69.5
*Marital status*		
Single	245	75.4
Married	71	21.8
Divorced/Widowed	9	2.8
*Number of family members*		
Less than 5 members	63	19.4
5–9 members	238	73.2
10 or more members	24	7.4
*Family income*		
Less than 500 USD	163	50.2
500–999 USD	136	41.8
1000–1499 USD	18	5.5
1500 USD or more	8	2.5
*Geographical region*		
Northern	203	62.5
Middle	112	34.5
Southern	10	3.1
*Living place*		
With family	309	95.1
University dormitory	7	2.2
With colleagues/friends	5	1.5
Alone	4	1.2
*Study program*		
Bachelor program	159	48.9
Bridging program (diploma-to-bachelor)	166	51.1
*Study year*		
1^st^ year	64	19.7
2^nd^ year	37	11.4
3^rd^ year	101	31.1
≥4^th^ year	123	37.8
*University*		
Governmental	105	32.3
Private	220	67.7
*Available Internet*		
Not available	6	1.8
Mobile data	138	42.5
Internet router	117	36.0
Two sources	64	19.7
*Available devices*		
Mobile phone	207	63.7
Desktop computer	8	2.5
Laptop	4	1.2
Tablet	3	0.9
More than one device	103	31.7

Outcome scores	Mean	Standard deviation

Item 1	3.04	1.24
Item 2	3.28	1.30
Item 3	2.49	1.19

Academic obstacles	2.94	0.95
Item 4	2.87	1.20
Item 5	3.12	1.16
Item 6	2.60	1.13
Item 7	3.49	1.16
Item 8	2.41	1.04

Technological obstacles	2.90	0.83
Item 9	3.14	1.19
Item 10	3.30	1.20
Item 11	3.30	1.19

Administrative obstacles	3.25	1.00
Overall obstacles' score	3.00	0.78

**Table 2:** Correlations among study variables (*N* = 325).

	(1)	(2)	(3)	(4)	(5)	(6)	(7)	(8)	(9)	(10)	(11)	(12)	(13)	(14)	(15)
(1) Gender	1.000														
(2) Marital status	0.136^*∗*^	1.000													
(3) No. of family members	0.082	−0.368^*∗∗*^	1.000												
(4) Family income	−0.016	−0.016	0.085	1.000											
(5) Geographical region	−0.090	−0.082	0.000	−0.030	1.000										
(6) Living place	−0.066	0.009	0.027	0.023	0.003	1.000									
(7) Study program	0.088	0.457^*∗∗*^	−0.201^*∗∗*^	−0.107	0.062	−0.001	1.000								
(8) Study year	0.103	0.003	0.075	0.100	−0.136^*∗*^	−0.047	0.076	1.000							
(9) University	−0.058	0.321^*∗∗*^	−0.141^*∗*^	−0.103	0.128^*∗*^	−0.082	0.653^*∗∗*^	−0.220^*∗∗*^	1.000						
(10) Available Internet	−0.039	−0.131^*∗*^	0.013	0.229^*∗∗*^	0.038	−0.097	−0.224^*∗∗*^	0.152^*∗∗*^	−0.262^*∗∗*^	1.000					
(11) Available devices	−0.094	−0.257^*∗∗*^	0.086	0.270^*∗∗*^	0.033	0.041	−0.381^*∗∗*^	0.081	−0.421^*∗∗*^	0.391^*∗∗*^	1.000				
(12) Academic obstacles	0.104	−0.172^*∗∗*^	0.065	0.102	0.015	0.096	−0.258^*∗∗*^	−0.027	−0.320^*∗∗*^	0.117^*∗*^	0.212^*∗∗*^	1.000			
(13) Technological obstacles	0.093	−0.165^*∗∗*^	0.071	−0.048	−0.058	0.157^*∗∗*^	−0.185^*∗∗*^	−0.072	−0.208^*∗∗*^	0.005	−0.001	0.570^*∗∗*^	1.000		
(14) Administrative obstacles	0.052	−0.260^*∗∗*^	0.104	0.036	−0.077	0.074	−0.274^*∗∗*^	0.041	−0.322^*∗∗*^	0.143^*∗∗*^	0.138^*∗*^	0.571^*∗∗*^	0.623^*∗∗*^	1.000	
(15) Overall obstacles' score	0.093	−0.237^*∗∗*^	0.079	0.017	−0.061	0.139∗	−0.277^*∗∗*^	−0.031	−0.326^*∗∗*^	0.087	0.124^*∗*^	0.804^*∗∗*^	0.888^*∗∗*^	0.840^*∗∗*^	1.000

^*∗*^ *p* < 0.05; ^*∗∗*^*p* < 0.01.

**Table 3:** Overall score differences based on participants' characteristics.

Variable	Group	*n*	Mean	SD	t/F	*P* value
Gender	Male	99	2.90	0.81	−1.62	0.111
	Female	226	3.05	0.77		

Study program	Regular	159	3.23	0.72	5.26	≤0.001
	Bridging	166	2.79	0.78		

University	Governmental	105	3.36	0.65	5.81	≤0.001
	Private	219	2.84	0.79		

Marital status	Single	245	3.12	0.74	10.63	≤0.001
	Married	71	2.66	0.80		
	Divorced/Widowed	9	2.72	1.03		

Number of family members	Less than 5 members	63	2.82	0.83	2.22	0.111
	5–9 members	238	3.05	0.76		
	10 or more members	24	3.01	0.85		

Family income	Less than 500 USD	163	3.01	0.80	1.29	0.278
	500–999 USD	136	2.96	0.75		
	1000–1499 USD	18	3.13	0.71		
	1500 USD or more	8	3.48	1.04		

Geographical region	Northern	203	3.04	0.78	1.03	0.358
	Middle	112	2.93	0.77		
	Southern	10	3.21	0.99		

Living place	With family	309	2.98	0.78	5.10	0.002
	University dormitory	7	3.95	0.61		
	With colleagues/friends	5	3.62	0.28		
	Alone	4	2.57	0.38		

Study year	1st year	64	2.99	0.87	0.74	0.532
	2nd year	37	3.18	0.85		
	3rd year	101	2.99	0.67		
	≥4th year	123	2.97	0.80		

Available Internet	Not available	6	3.94	1.02	1.03	0.36
	Mobile data	138	2.90	0.79		
	Internet router	117	2.98	0.71		
	Two sources	64	3.20	0.81		

Available devices	Mobile phone	207	2.95	0.82	1.61	0.173
	Desktop computer	8	2.73	0.90		
	Laptop	4	2.57	0.53		
	Tablet	3	3.27	0.24		
	More than one device	103	3.14	0.71		

**Table 4:** Mann–Whitney test for subscale differences based on participants' characteristics.

Characteristics	Outcome variable	Group	*N*	Mean rank	Mann–Whitney U	Z	*P* value
Gender	Academic obstacles	Male	99	148.30	9732	−1.88	0.060
		Female	226	169.44			
	Technological obstacles	Male	99	149.89	9889	−1.67	0.095
		Female	226	168.74			
	Administrative obstacles	Male	99	155.74	10486.5	−0.93	0.354
		Female	226	166.18			

Study program	Academic obstacles	Regular	159	187.56	9291.5	−4.64	≤0.001
		Bridging	166	139.47			
	Technological obstacles	Regular	159	180.65	10390.5	−3.32	0.001
		Bridging	166	146.09			
	Administrative obstacles	Regular	159	189.14	9040.5	−4.94	≤0.001
		Bridging	166	137.96			

University	Academic obstacles	Governmental	105	205.42	6990.5	−5.75	≤0.001
		Private	219	141.92			
	Technological obstacles	Governmental	105	190.47	8560.5	−3.73	≤0.001
		Private	219	149.09			
	Administrative obstacles	Governmental	105	205.67	6964.5	−5.78	≤0.001
		Private	219	141.80			

**Table 5:** Kruskal–Wallis tests for subscale differences based on participants' characteristics.

Characteristics	Outcome variable	Group	*N*	Mean rank	*X* ^2^	*P* value
Gender	Academic obstacles	Single	245	172.17	9.60	0.008
		Married	71	135.27		
		Divorced/Widowed	9	132.11		
	Technological obstacles	Single	245	172.08	9.97	0.007
		Married	71	132.24		
		Divorced/Widowed	9	158.61		
	Administrative obstacles	Single	245	176.96	22.27	≤0.001
		Married	71	119.68		
		Divorced/Widowed	9	124.78		

Number of family members	Academic obstacles	Less than 5 members	63	140.29	7.123	0.028
		5–9 members	238	171.36		
		10 or more members	24	139.71		
	Technological obstacles	Less than 5 members	63	149.38	1.747	0.417
		5–9 members	238	165.71		
		10 or more members	24	171.92		
	Administrative obstacles	Less than 5 members	63	141.29	4.234	0.120
		5–9 members	238	168.04		
		10 or more members	24	170.04		

Family income	Academic obstacles	Less than 500 USD	163	155.98	6.404	0.094
		500–999 USD	136	163.33		
		1000–1499 USD	18	210.03		
		1500 USD or more	8	194.63		
	Technological obstacles	Less than 500 USD	163	168.94	4.521	0.210
		500–999 USD	136	154.22		
		1000–1499 USD	18	152.53		
		1500 USD or more	8	214.81		
	Administrative obstacles	Less than 500 USD	163	160.83	1.085	0.781
		500–999 USD	136	162.19		
		1000–1499 USD	18	178.50		
		1500 USD or more	8	186.13		

Geographical region	Academic obstacles	Northern	203	162.47	1.298	0.523
		Middle	112	161.02		
		Southern	10	195.90		
	Technological obstacles	Northern	203	167.53	1.576	0.455
		Middle	112	154.05		
		Southern	10	171.35		
	Administrative obstacles	Northern	203	169.46	5.335	0.069
		Middle	112	148.07		
		Southern	10	199.15		

Living place	Academic obstacles	With family	309	160.84	18.195	≤0.001
		University dormitory	7	285.43		
		With colleagues/friends	5	207.50		
		Alone	4	60.00		
	Technological obstacles	With family	309	159.63	10.230	0.017
		University dormitory	7	239.36		
		With colleagues/friends	5	258.10		
		Alone	4	170.88		
	Administrative obstacles	With family	309	161.34	10.884	0.012
		University dormitory	7	254.21		
		With colleagues/friends	5	204.00		
		Alone	4	80.25		

Study year	Academic obstacles	1st year	64	162.54	3.125	0.373
		2nd year	37	187.27		
		3rd year	101	155.76		
		≥4th year	123	161.89		
	Technological obstacles	1st year	64	166.98	2.323	0.508
		2nd year	37	176.57		
		3rd year	101	167.00		
		≥4th year	123	153.57		
	Administrative obstacles	1st year	64	154.15	2.928	0.403
		2nd year	37	179.09		
		3rd year	101	155.16		
		≥4th year	123	169.20		

Available Internet	Academic obstacles	Not available	6	213.83	8.769	0.033
		Mobile data	138	149.82		
		Internet router	117	162.78		
		Two sources	64	187.05		
	Technological obstacles	Not available	6	275.92	10.862	0.012
		Mobile data	138	158.62		
		Internet router	117	155.61		
		Two sources	64	175.38		
	Administrative obstacles	Not available	6	218.58	12.882	0.005
		Mobile data	138	147.86		
		Internet router	117	161.02		
		Two sources	64	194.05		

Available devices	Academic obstacles	Mobile phone	207	148.52	17.574	0.001
		Desktop computer	8	170.38		
		Laptop	4	107.38		
		Tablet	3	223.17		
		More than one device	103	191.93		
	Technological obstacles	Mobile phone	207	163.64	2.671	0.614
		Desktop computer	8	134.25		
		Laptop	4	111.00		
		Tablet	3	206.33		
		More than one device	103	164.71		
	Administrative obstacles	Mobile phone	207	154.66	10.831	0.029
		Desktop computer	8	117.38		
		Laptop	4	111.50		
		Tablet	3	153.67		
		More than one device	103	185.58		

## Data Availability

The data used to support the findings of this study are available from the corresponding author upon request.
